# Anaerobic adaptation of *Mycobacterium avium* subspecies *paratuberculosis* in vitro: similarities to *M. tuberculosis* and differential susceptibility to antibiotics

**DOI:** 10.1186/s13099-017-0183-z

**Published:** 2017-06-10

**Authors:** Nicole Parrish, Aravinda Vadlamudi, Neil Goldberg

**Affiliations:** 1 The Johns Hopkins Medical Institutions, 600 North Wolfe Street, Meyer B1-193, Baltimore, Maryland USA; 2 Saint Joseph Medical Center, University of Maryland, Towson, Maryland USA

## Abstract

**Background:**

*Mycobacterium avium* subspecies *paratuberculosis* (MAP) is the causative agent of Johne’s disease in ruminants and is associated with Crohn’s disease (CD) in humans, although the latter remains controversial. In this study, we investigated the ability of MAP to adapt to anaerobic growth using the “Wayne” model of non-replicating persistence (NRP) developed for *M. tuberculosis*.

**Results:**

All strains adapted to anaerobiosis over time in a manner similar to that seen with MTB. Susceptibility to 12 antibiotics varied widely between strains under aerobic conditions. Under anaerobic conditions, no drugs caused significant growth inhibition (>0.5 log) except metronidazole, resulting in an average decrease of ~2 logs.

**Conclusions:**

These results demonstrate that MAP is capable of adaptation to NRP similar to that observed for MTB with differential susceptibility to antibiotics under aerobic versus anaerobic conditions. Such findings have significant implications for our understanding of the pathogenesis of MAP in vivo and the treatment of CD should this organism be established as the causative agent.

## Background

Crohn’s disease (CD) is an incurable, chronic inflammatory disorder of the gastrointestinal tract [1]. Although the etiology of CD is unknown, the clinical findings in humans resemble those of Johne’s disease (JD) in cattle, caused by *Mycobacterium avium* subspecies *paratuberculosis* (MAP) [1]. In cattle, MAP was established as the etiologic agent of JD by successful demonstration of Koch’s postulates [2]. In humans, MAP as the causative agent of CD has been met with both support and skepticism despite the similarities to JD [3]. Supporting evidence for MAP in the etiology of CD may be found in multiple studies in which the organism has either been cultured from intestinal tissues, breast milk, and the blood of CD patients or MAP DNA/RNA has been detected in patient samples versus healthy controls [4–10]. Exposure to MAP may be more widespread than is recognized since viable organism has been found in potable water, commercial milk, and other dairy products, including those having undergone pasteurization sufficient to kill common contaminating organisms [4, 8, 11–13]. MAP can also persist in the environment for long periods of time in the absence of a host as evidenced by pastures which remain infective for months after removal of all infected animals [14, 15]. Unfortunately, the time required for clearance of MAP in the environment is largely unknown.

Skepticism of MAP as the causative agent of CD stems from several lines of evidence which support a strong role for immune dysregulation and highlight failure to achieve a cure with antimicrobial therapy [1, 16]. Antimicrobial regimens for treatment of CD have included antibiotics such as rifaximin (RFX), ciprofloxacin (CIP), and metronidazole (MET) given separately or in combination for a period of one to several months [18–22]. Other trials have used clarithromycin, clofazimine or rifabutin [23]. Efficacy is variable and relapse a common occurrence once therapy is stopped. Questions surrounding the cause of this relapse remain unanswered. Some investigators have suggested the possibility that MAP may be capable of entering a dormant or non-cultivable state in which reversion to vegetative growth is possible when more favorable environmental conditions are present [14]. Dormancy (also known as ‘latency’) is well documented with respect to other mycobacterial species such as *M. tuberculosis* and *M. bovis* BCG (BCG) [24–26]. These related species possess the ability to transition to a non-replicating ‘latent’ or persistent state in which metabolism is reduced to an extremely low basal level as part of an adaptive response to anaerobiosis [25, 26]. These ‘latently adapted’ organisms can persist for years or decades until reactivation occurs due to a variety of factors including waning of immunosurveillance. Recently, some investigators noted that although most humans appear to be susceptible to MAP infection, few develop clinical signs and symptoms immediately following exposure. The authors postulate that in these individuals a ‘latent’ rather than an ‘active’ infection is established; an infection which is controlled rather than eliminated by the immune response [27]. Transition of MAP from a vegetative to a ‘latent’ state may require environmental signals, such as occurs with *M. tuberculosis* in response to decreasing oxygen concentrations. Once adapted to the ‘latent’ or non-replicating persistent state, MAP may exhibit differential susceptibility to various antibiotics as has been documented with *M. tuberculosis*. In culture, latently adapted *M. tuberculosis* is not susceptible to the majority of commonly used first-line antimycobacterial drugs; suggesting that this population of bacilli cannot be eliminated by conventional antimicrobial therapy which relies on actively growing bacilli to be effective. Only metronidazole (MET), active in anaerobic but not aerobic conditions, significantly inhibits this population of *M. tuberculosis* in vitro [25, 28]. If MAP were capable of anaerobic adaptation to a non-replicating persistent state as seen in *M. tuberculosis* and BCG, then most anti-mycobacterial drug regimens would be insufficient to eradicate this population of organisms [24–26, 28]. Interestingly, metronidazole has shown some efficacy in the treatment of Crohn’s disease [17–19].

The goal of this study was aimed at answering two specific questions related to the biology of MAP: (1) this fastidious species is known to grow under aerobic conditions so long as culture medium is supplemented with Mycobactin J; however, can MAP adapt to a ‘latent’ or non-replicating, persistent state in vitro as is the case with *M. tuberculosis* and BCG and, (2) if so, what is the susceptibility (in vitro) of MAP to antimycobacterial drugs under aerobic versus anaerobic conditions?

## Methods

### Mycobacterial strains and maintenance conditions

Five strains of MAP were used in this study including one control strain (19,698, type strain, American Type Culture Collection, ATCC, Rockville, MD), three bovine derived strains designated B1 through B3, and one human associated strain (Ben, ATCC 43544). *M. bovis* BCG (Pasteur, ATCC 35734) was used as a control for all assays as this organism has been well characterized in the Wayne model of non-replicating persistence. All strains were maintained on Herrold’s egg yolk agar slants containing 2.0 µg/ml Mycobactin J (Becton–Dickinson, Sparks, Maryland) at 37 °C in an atmosphere of 5% CO_2_.

### Aerobic susceptibility testing

Aerobic susceptibility testing was conducted using broth microdilution in a 96-well plate format. Briefly, a suspension of each strain to be tested was prepared in Middlebrook 7H9 (M7H9) broth (Difco, Detroit, Michigan) supplemented with 2.0 µg/ml Mycobactin J (Allied Monitor, Fayette, Missouri) and diluted to a final density of ~10^5^ CFU/ml. Plates were inoculated with 100 µl of the adjusted suspension and the supplied cover put in place, with subsequent incubations for up to 14 days at 37 °C. All plates were read manually using a mirror box and ambient light. The minimum inhibitory (MIC_99_) concentration was determined by comparing growth in the control wells to growth in antibiotic containing wells; the lowest concentration of each drug tested resulting in 99% inhibition versus the untreated controls was interpreted as the MIC. Antimicrobial agents and their concentrations tested included AMI (1–64 µg/ml), GEN (0.5–16 µg/ml), CLR (0.125–16 µg/ml), EMB (0.5–32 µg/ml), MES (1.5–25 µg/ml), SAL (1.5–25 µg/ml), RIF (0.12–16 µg/ml), RFX (0.12–16 µg/ml), CIP (0.12–4 µg/ml), and MET (12.5 µg/ml). All antimicrobial agents with one exception were obtained from Sigma-Aldrich, St. Louis, Missouri. RFX was supplied by Salix Pharmaceuticals, Raleigh, North Carolina. All assays were performed in triplicate and purity plates were done for each susceptibility test.

### Anaerobic studies

We used the in vitro Wayne model of NRP to determine the ability of MAP to survive under anaerobic conditions [25, 26]. Briefly, MAP cultures were grown in Dubos Tween-albumin broth supplemented with Mycobactin J (2.0 µg/ml). Oxygen was gradually depleted from aerobic, exponentially growing cultures (~10^6^ CFU/ml) by aeration at 250 rpm’s for a period of approximately 10–12 days using Hungate-type anaerobic culture tubes. Optical density and colony forming units were determined to monitor the progression of the cultures from aerobic growth through NRP stages 1 and 2. Methylene blue was used to detect the presence of oxygen in the culture. Complete decolorization was used as an indicator for anaerobiosis. Once anaerobiosis had been established, varying concentrations of each antibiotic were added to respective test cultures at concentrations equivalent to as well as several-fold above the aerobic MIC: AMI (up to 16 µg/ml), GEN (up to 16 µg/ml), CLR (up to 8 µg/ml), EMB (up to 32 µg/ml), RIF (up to 8 µg/ml), RFX (up to 8 µg/ml), and CIP (up to 8 µg/ml). This was followed by further incubation for an additional 48 h. MES and SAL were not tested in the anaerobic model as they failed to inhibit any growth in the aerobic assay. BCG was tested in parallel as a control to demonstrate that the anaerobic model was performing as expected using isoniazid (INH: 0.1–0.4 µg/ml) and RIF (0.06–0.1 µg/ml) since neither drug is effective in killing BCG in the anaerobic model [26]. MET (12.5 µg/ml), which was tested at a single concentration, was used as a positive control since it is only active under anaerobic conditions [25, 26]. For each culture, serial dilutions were made and plated to M7H10 agar. Following ~15 days of incubation, the CFU/ml were determined for each culture condition and compared to the untreated and positive and negative controls. All assays were performed in triplicate.

## Results

### Aerobic growth and antibiotic susceptibilities

Aerobic susceptibilities varied widely between strains for most of the antibiotics tested (Table 1). Minimum inhibitory concentrations (MIC’s, 99% inhibition) were most consistent between strains with ciprofloxacin (CIP, range 1–2 µg/ml). However, greater heterogeneity in MIC’s was noted between MAP strains with the remaining active drugs: rifaximin (RFX, 0.25–1), rifampin (RIF, 0.25–4), amikacin (AMI, 2–8), clarithromycin (CLR, 0.125–2), ethambutol (EMB, 2–16), and gentamicin (GEN, 1–4). No inhibition was seen in any MAP strains exposed to metronidazole (MET), mesalamine (MES), or salicylin (SAL) under aerobic conditions at the highest concentration tested for each drug (12.5, 25 and 25 µg/ml, respectively).

**Table 1:** Minimum inhibitory concentrations to various antibiotics for MAP strains used in this study under aerobic versus anaerobic conditions

MAP strain	Condition	Test ranges and MICs (µg/ml)									
		RIF	RFX	AMI	CIP	CLR	EMB	GEN	MET	MES	SAL
ATCC 19698	Aerobic	2	0.5	2	2	0.5	4	1	>12.5	>25	>25
	Anaerobic	>8	>8	>16	>8	>8	>16	>16	12.5	NT	NT
B-1	Aerobic	1	1	4	2	1	8	2	>12.5	>25	>25
	Anaerobic	>8	>8	>16	>8	>8	>16	>16	12.5	NT	NT
B-2	Aerobic	0.25	0.25	2	1	0.125	2	2	>12.5	>25	>25
	Anaerobic	>8	>8	>16	>8	>8	>16	>16	12.5	NT	NT
B-3	Aerobic	2	0.5	4	2	2	16	2	>12.5	>25	>25
	Anaerobic	>8	>8	>16	>8	>8	>16	>16	12.5	NT	NT
ATCC 43544	Aerobic	4	1	8	1	1	8	4	>12.5	>25	>25
	Anaerobic	>8	>8	>16	>8	>8	>16	>16	12.5	NT	NT

The concentrations shown are the highest tested for each antibiotic in the anaerobic model

*M. bovis* BCG ATCC 35734 (Pasteur) was used as a control for the anaerobic model as previously described [45]. Aerobic MICs for BCG were <0.1 for isoniazid and ≤0.06 for rifampin. Neither drug significantly inhibited growth of BCG in the anaerobic model at concentrations above (INH >0.4; RIF >0.1) that observed under aerobic conditions

*RIF* rifampin, *RFX* rifaximin, *AMI* amikacin, *CIP* ciprofloxacin, *CLR* clarithromycin, *EMB* ethambutol, *GEN* gentamicin, *MET* metronidazole, *MES* mesalamine, *SAL* salicilin, *NT* not tested

### Anaerobic adaptation and antibiotic susceptibilities

**Fig. 1:**
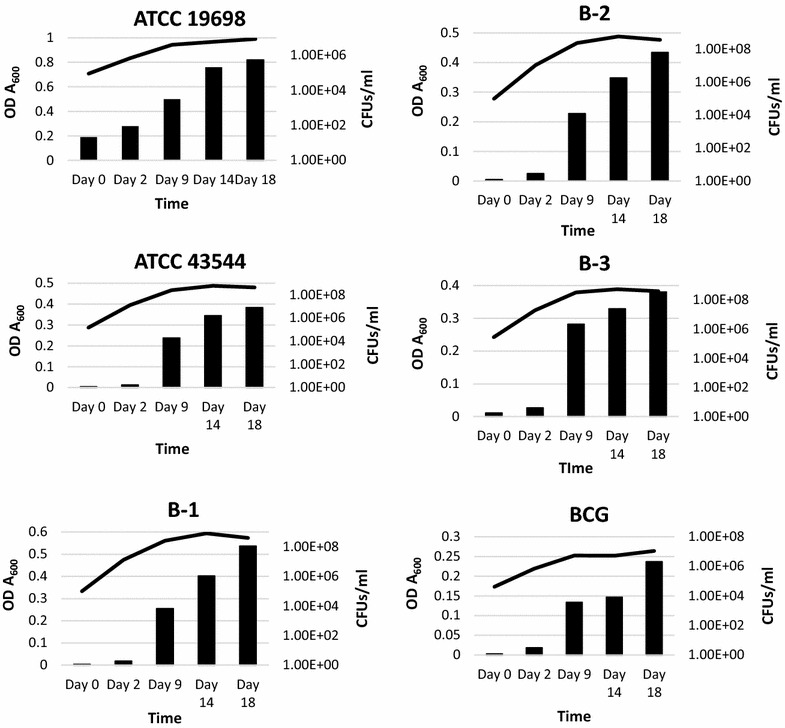
Growth and adaptation of MAP versus *M. bovis* BCG in the in vitro Wayne model of anaerobiosis. *CFU/ml* colony forming units per ml of culture, *OD* optical density of broth culture when read at A_600_ nm. *Lines indicate* CFUs/ml, *bars indicate* OD A_600_. Data shown for MAP strains ATCC 19698 and 43544 as well as B-1, B-2, B-3, and BCG (*M. bovis* BCG ATCC 35734). Fading of methylene blue occurred for all strains tested between 12 and 14 days; complete decolorization occurred for all strains between days 18 and 19 indicating anerobiosis. All strains tested exhibited a continued increase in OD A_600_ at the same point in time at which CFUs were leveling off consistent with adaptation to anaerobiosis and non-replicating persistence

All strains demonstrated adaptation to anaerobiosis similar to that of *M. tuberculosis* and BCG with growth ranging from ~10^7^ to 10^8^ CFU/ml after ~10–14 days incubation from a starting inoculum of ~10^6^ CFU/ml. As shown in Fig. 1, optical density (OD) and colony forming units (CFU’s) increased in parallel until ~day 10. At this time, CFU’s began to level off and the methylene blue began to fade (days 12 through 16). This stage was followed by a slight increase in OD’s without concomitant increase in CFU’s/ml. For all strains, by days 18–19, the methylene blue had completely faded indicating conversion to anaerobiosis. Anaerobic susceptibilities indicated more homogeneous results than those observed with aerobically growing cultures. No drugs tested resulted in 99% inhibition of growth; thus an MIC_99_ could not be established under anaerobic conditions (Table 1). Only MET (12.5 µg/ml) showed appreciable activity with a 2-log_10_ drop in CFU’s/ml following 48 h exposure. Of the other drugs tested, only RIF, and RFX showed minimal activity with 0.5 log_10_ inhibition of growth. However, inhibition with RIF and RFX required the use of concentrations 1- to 8-fold above the MIC for each drug compared with aerobically growing cultures. No activity was observed with the remaining drugs: AMI, CIP, CLR, EMB, and GEN with <0.5 log decrease in CFU/ml. MES and SAL were not tested in the anaerobic model as they were not active against aerobically growing strains.

## Discussion

The results from this study demonstrate that MAP is capable of both aerobic growth and adaptation in anaerobiosis when using the ‘Wayne’ in vitro model developed for *M. tuberculosis*. This particular model illustrates the progression of *M. tuberculosis* through two stages of non-replicating persistence (NRP) in response to the gradual withdrawal of oxygen from a culture over a period of days to weeks. Gradual withdrawal of oxygen permits time for genetic and phenotypic adaptation to anoxic conditions via progression through two stages of NRP [25]. NRP-1 is a microaerophilic state in which culture OD’s increase, although at a slower rate than during vegetative growth, without a concomitant increase in CFU’s/ml. NRP-2 is characterized by adaptation to complete anaerobiosis with no further increase in OD [25]. In the current study, not only did MAP demonstrate clear progression though both NRP-1 and NRP-2, but also in much the same manner as that observed with *M. tuberculosis*. It has been suggested that the ability of *M. tuberculosis* to adapt to NRP-1 and NRP-2 is responsible for the survival of the organism over long periods of time in the human host. Reversion to a vegetative state occurs when conditions are more favorable leading to reactivation of ‘latent’ disease. In this way, *M. tuberculosis* is able to persist in tissues for months to years without replicating or causing active disease while remaining immunologically antigenic. Recently, Janagama et al. examined the transcriptome of MAP in vivo and found that transcripts pertaining to latency were upregulated in intestinal tissues indicating that regulation of these specific pathways appear to be related to tissue and cell type [29]. They showed that MAP obtained from tissues exhibited significant “shutdown” of major metabolic pathways suggesting that MAP survival was directly correlated with the ability to evade the immune response by entering a persistent state and surviving within macrophages [29, 30]. Investigators have also noted that most humans appear to be susceptible to MAP infection; however, few develop clinical signs and symptoms immediately following exposure [16, 31–34]. Although the reasons for this delay have not been elucidated, one possibility is that any MAP present have entered a state of NRP and much like *M. tuberculosis*, establish a ‘latent’ infection which the immune response is unable to eliminate. Waning of immune surveillance would necessarily lead to a reversal of NRP and potential development of active disease. Further complicating the picture in CD is the presence of polymorphisms in the *NOD2/CARD15* gene which have been identified in those with the disease versus paired controls [35]. Not only may *NOD2/CARD15* play a role in the defense against pathogenic bacteria, but more importantly provide for regulation of mediators of intestinal inflammation. The presence of anaerobically adapted MAP in intestinal tissues may lead to persistent antigenic stimulation in the absence of cultivable bacilli not unlike that observed in those with latent *M. tuberculosis* infection. Recently, a large meta-analysis was performed which investigated genome-wide association scans of over 75,000 cases/controls of CD and ulcerative colitis (UC) patients. This study found a strong association between CD/UC with genes involved in primary immunodeficiencies. Interestingly, a subset of the genes involved in primary immunodeficiencies was also associated with increased susceptibility to mycobacterial infection suggesting that those with CD/UC may be more susceptible to not only MAP but also *M. tuberculosis* as well [36].

Further similarities between MAP and *M. tuberculosis* were demonstrated in the present study by the differential susceptibility to antibiotics observed under aerobic versus anaerobic conditions. When completely adapted to anaerobiosis, *M. tuberculosis* is “impervious” to the activity of most aerobically active drugs as seen with MAP strains tested in this study [25, 28]. Only MET exhibited any activity against anaerobically adapted MAP strains, findings consistent with those observed for *M. tuberculosis* in the same model [25]. MET, is a prodrug which requires activation under anaerobic conditions [25]. Thus, the lack of activity observed for MET with aerobically growing MAP strains is consistent with the known mechanism of action of this drug. In addition, the log_10_ reduction in CFU’s/ml observed in MET exposed MAP under anaerobic conditions closely mirrored that seen with BCG, used as a control in this study.

This study does have some limitations. First, MES failed to inhibit any of the MAP strains tested in this study when grown under aerobic conditions; thus this drug was not tested in the anaerobic model. However, previous investigators reproducibly demonstrated weak bacteriostatic activity of MES against aerobically adapted MAP using the sensitive BACTEC 460^®^ radiometric system (Becton–Dickinson, Sparks, MD) [37, 38]. Interestingly, the same results were not obtained using the BACTEC Mycobacterial Growth Indicator Tube (MGIT-960^®^, Becton–Dickinson) [37–39]. It is possible that differences in testing methods are responsible for the variability in MAP susceptibility to MES observed between studies. Unfortunately, the most sensitive of the methods used for MAP susceptibility testing, the BACTEC 460 system, is no longer commercially supported nor are consumables available, which in particular negatively impacts assessment of bacteriostatic drugs. Secondly, a number of investigators have demonstrated dose-dependent inhibition of MAP in culture by a number of anti-inflammatory drugs including methotrexate and 6-mercaptopurine [37–44]. As a result, Greenstein et al. postulated that treatment of patients with inflammatory bowel disease with methotrexate and 6-mercaptopurine may result in inhibition of MAP and secondarily a decrease in pro-inflammatory cytokines [42]. In the current study, testing of anti-inflammatory drugs was beyond the scope of this project. However, future studies are planned which will determine the activity of anti-inflammatory drugs against MAP under both aerobic and anaerobic conditions.

Although a definitive causal link between CD and MAP has not been established, the findings as presented in this investigation raise some interesting questions. Could the presence of anaerobically adapted MAP in intestinal tissues lead to persistent antigenic stimulation and the chronic inflammation observed in CD? Could the variable efficacy of different antibiotic regimens used in CD patients be related to the presence of mixed populations of aerobically and anaerobically adapted MAP? Is relapse related to ‘reactivation’ of anaerobically adapted organisms not eliminated by conventional antibiotic regimens? Could this explain why some studies have shown efficacy in CD patients receiving MET? These and other questions require further investigation including prospective, randomized, clinical trials which utilize antimicrobial combination regimens active against both aerobic and anaerobically adapted MAP.

## Conclusions

This study demonstrates the ability of MAP to adapt to anaerobiosis in the in vitro Wayne model developed for *M. tuberculosis*. Adaptation of MAP to a non-replicating persistent state demonstrated differential susceptibility to antibiotics under aerobic versus anaerobic conditions. This has implications for treatment to eradicate this organism in vivo as standard antibiotics currently used to target MAP have little to no effect against non-replicating persistent bacilli. Since mixed populations of MAP are likely to exist simultaneously in vivo, combination therapies containing metronidazole and aerobically active drugs such as clarithromycin and rifaximin should be considered.
